# Numerical Simulation Study on Fish Habitats in the Downstream Section of Yangqu Hydropower Station

**DOI:** 10.1002/ece3.70756

**Published:** 2025-01-16

**Authors:** Qiaoling Zhang, Youjie Ou, Weiying Wang, Guoyong Zhang, Zijun Liu, Shanshan Li, Guodong Li

**Affiliations:** ^1^ State Key Laboratory of eco‐Hydraulics in Northwest Arid Region of China Xi'an University of Technology Xi'an China; ^2^ China Renewable Energy Engineering Institute Beijing China

**Keywords:** ecological flow, fish weighted habitat area, fuzzy logic method, habitat quality, Yellow River

## Abstract

To promote the coordinated and sustainable development of hydropower exploitation and ecological environment in the upper reaches of the Yellow River, a fine simulation of the downstream riverway of Yangqu Hydropower Station was carried out to analyze the impact of the changes in water depth and flow velocity on fish habitats after the impoundment of Yangqu Hydropower Station. In this paper, *Gymnocypris eckloni* was selected as the target fish species. The fish habitat model was constructed using MIKE21. The habitat quality of the target fish species was graded by the fuzzy logic method with suitable flow velocity and water depth as conditions. The Weighted Usable Area (WUA)—flow response relationship of fish habitats under different qualities was studied, and the ecological flow required by the target fish species was obtained. The results indicated that the suitable ecological flow range, derived from the relationship between the weighted total area of habitats of different qualities and flow variations, ranged from 350 to 1100 m^3^/s. Furthermore, the suitable flow range determined through the proportion of WUA of habitats of different qualities was between 600 and 1150 m^3^/s. After a comprehensive analysis, the final suitable ecological flow range was determined to be 600 to 1150 m^3^/s. The proportion of high‐quality habitat WUA ranged from 0.26 to 0.50, As the traffic increased, it first increased and then decreased, and was most affected by changes in traffic, the proportion of medium‐quality habitat WUA fluctuated between 0.40 and 0.55, showing an overall upward trend. Meanwhile, the proportion of low‐quality habitat WUA increased from 0.11 to 0.21, indicating the smallest impact from flow variations. The research results could provide a certain reference for the ecological scheduling of hydropower stations in the upper reaches of the Yellow River.

## Graphical abstract

Study on fish habitats in the downstream river of Yangqu Hydropower Station found that high‐quality habitats are most sensitive to flow changes. The most suitable ecological flow range is 600–1150 m^3^/s, vital for maintaining ecological stability and fish conservation in the Yellow River.

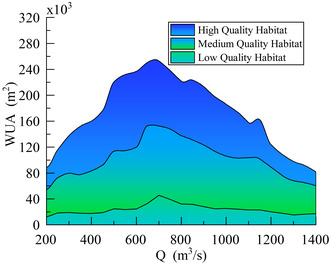

## 1 Introduction

The dam across the river played an important role in flood control, power generation, water supply, and shipping (Boets et al. 2018; Guo et al. 2023). However, to a certain extent, it also altered the natural runoff process of the Yellow River, causing river cutoff, destroying fish habitats, and affecting the river's ecological environment (Vörösmarty et al. 2010; Wang, Chen et al. 2021; Ahmed et al. 2022). The deterioration of the ecological environment upstream of the Yellow River led to water shortages and flow disruptions, resulting in a shortage of freshwater dilution and nutrient sources for the ocean, posing a serious threat to the marine ecological environment. To protect the ecosystems of rivers and oceans, we established a connection between fish habitats and changes in river flow, using changes in river flow as a condition and changes in fish habitats as a criterion for evaluating the ecosystem (Jing 2013; Mishra et al. 2022). This allowed us to obtain the ecological flow required to meet the reproductive needs of fish (Wang, Han et al. 2023).

At present, the methods for calculating river ecological flow mainly include hydrological methods, hydraulic methods, and habitat simulation methods (Tharme 2003; Acreman and Dunbar 2004). The hydrological and hydraulic methods were limited due to their lack of a biological basis, whereas the habitat simulation methods comprehensively considered factors such as flow velocity, water depth, and river sediment. These methods established a closer relationship between biological habits and habitat conditions, enabling a more comprehensive and objective evaluation of habitat environments. Consequently, they were widely applied in river ecological management (Kripa et al. 2018; Yu et al. 2021). The method typically employed the Instream Flow Incremental Methodology (IFIM) or the Physical Habitat Simulation Model (PHABSIM). It established the relationship between flow rate and habitat area of target species through the Weighted Usable Area (WUA), providing a scientific basis for maintaining ecological stability (Stalnaker, Chisholm, and Paul 2017; Sharma et al. 2022). Considering the influence of flow velocity, water depth, river sediment, and other factors, the relationship between river flow and the habitat area of target species was established, which could be used as an important index to maintain river ecological stability. Cai et al. (2024) simulated the effects of ecological spur dikes on meandering rivers by combining a two‐dimensional water‐sediment coupled shallow water equation with the IFIM. Miao et al. (2020) integrated the WUA calculated by the PHABSIM model with ecological scheduling to determine the minimum and suitable ecological demands for the river. However, the PHABSIM model did not sufficiently account for river inflow. As a one‐dimensional river simulation method, it was generally applicable only to habitat simulation studies of river cross‐sections or specific river sections, making it difficult to fully capture complex flow conditions and unable to accurately simulate more complex river sections (Shan et al. 2022).

After Alberta University combined the River‐2D model with the IFIM method, a new habitat simulation approach was established. The integration of numerical models at different scales with habitat simulation methods became increasingly widespread, with studies incorporating models such as Hydrologic Engineering Center’s River Analysis System (HEC‐RAS), MIKE11, MIKE21, and HABIOSIM (Yi et al. 2017; Tang et al. 2021). Zhu et al. (2020) used the HEC‐RAS model to generate one‐dimensional hydrodynamic results as boundary conditions and then applied the River‐2D model for two‐dimensional hydrodynamics and fish habitat simulation to assess the effectiveness of various conservation measures in restoring habitats. Chen et al. (2023) coupled the MIKE21 model with a habitat model to establish a fish habitat model for the upper Yangtze River, quantifying the impact of flow variations on river habitats. Lee, Kil, and Jeong (2010) used the River‐2D model to analyze the hydraulic characteristics of fish habitats in urban rivers and compared them to traditional methods of artificial fish habitat creation. The two‐dimensional hydrodynamic‐based habitat simulation method enabled more precise modeling of hydraulic features within habitats, reflecting changes in flow patterns and improving computational accuracy, thus providing more accurate guidance for river ecological research and management.

In the research process, most methods directly calculate the total WUA of the target river section for habitat suitability evaluation. However, these approaches often fail to adequately consider the varying habitat quality requirements of fish species. This oversight can lead to potential biases in species conservation efforts (Boets et al. 2018; Kripa et al. 2018; Wang, Wan et al. 2021). Therefore, researchers introduced methods such as fuzzy logic, multiple linear regression, and landscape ecological indicators into fish habitat models (Austin 2007). Wang, Deng et al. (2023); using long‐term hydrological data, constructed a habitat suitability evaluation index system and assessment standards through fuzzy logic, revealing the temporal and spatial dynamics of fish habitat suitability. Li et al. (2015) combined fuzzy logic and landscape ecology methods to develop an ecological flow model based on fish habitat suitability. They established a relationship between flow and both the quantity and quality of habitats. These methods evaluated the quality of habitat areas based on the innate preferences of target species, aiming to improve the accuracy of the models in practical applications (Hu et al. 2020). Among them, fuzzy logic was widely used in fish habitat preference analysis and fish habitat quality evaluation, as it could numerically process qualitative information on fish species' innate preferences and consider the correlation between multiple variables.

The study area was located upstream of the Yellow River basin, which was one of the important spawning grounds for fish (Zhang et al. 2023). However, the construction of dams altered the original flow conditions of the river, restricting the fish's ability to freely select habitats for foraging and spawning, thus changing the hydrological conditions of the river (Lin et al. 2024; Simmons et al. 2024). Additionally, difficulties in terrain surveying and the absence of long‐term monitoring data led to relatively limited research on fish habitats in this region. These factors made it particularly important to understand and protect the fish habitats upstream of the Yellow River, necessitating further research to reveal their ecological characteristics and patterns of change.

Based on this, the present study selected the downstream river section of the Yangqu Hydropower Station as the research area, with *Gymnocypris eckloni* as the target fish species. The MIKE21 hydrodynamic model was coupled with a fish habitat model to calculate the suitability distribution of the target fish species within the study area. The fuzzy logic method was applied to classify the habitat quality, and an analysis was conducted to determine the weighted suitability area and proportion of fish habitats with different qualities. A response relationship was established between the WUA and the proportion of habitats with different qualities and the flow rate, considering the needs of fish for different habitat qualities. Based on this comprehensive analysis, the ecological flow range required by the target fish species was determined. The research results can provide basic data for maintaining ecological stability and fish conservation in the upper reaches of the Yellow River.

## 2 Materials and Methods

### 2.1 Study Area

**FIGURE 1:**
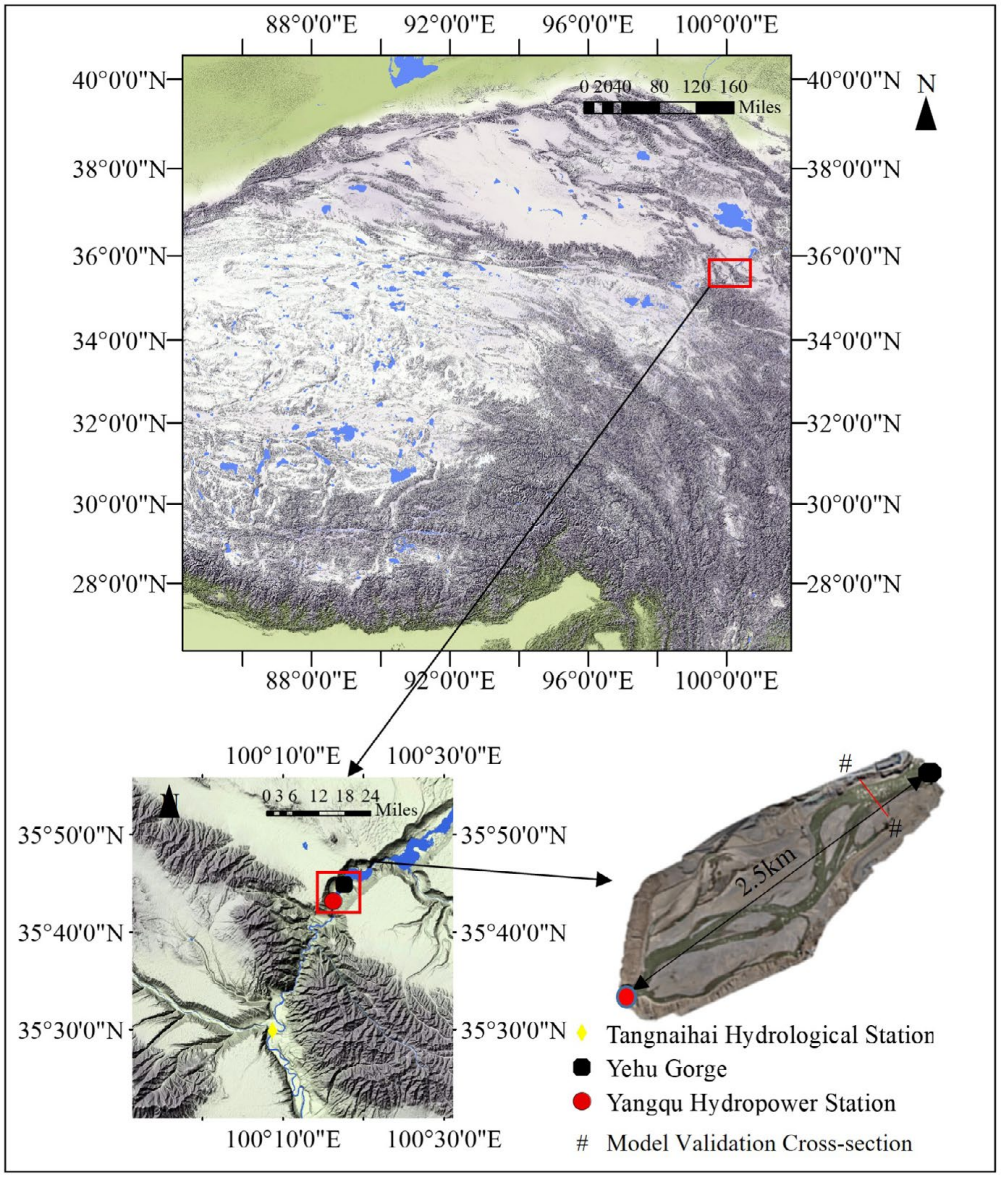
Study the river section.

The Yangqu Hydropower Station was located at the Yangqu Gorge, which was the border between Xinghai County and Guinan County in Hainan Prefecture, Qinghai Province. It was a large‐scale water control project primarily for power generation and was part of the planned cascade of hydropower stations upstream of the Longyangxia Hydropower Station on the main stem of the Yellow River (Li et al. 2018; Jia et al. 2020). The study area was situated downstream of the Yangqu Hydropower Station in the upper reaches of the Yellow River, with a total length of approximately 2.5 km. It belonged to a wide‐valley type river section, with the upstream being the Yangqu Hydropower Station and the downstream being the Yehuxia Gorge. The river terrain in this region was complex, characterized by a large area of shallow water areas and variable water flow patterns. The study area is shown in Figure 1.

### 2.2 Selection of Target Fish Species

**FIGURE 2:**
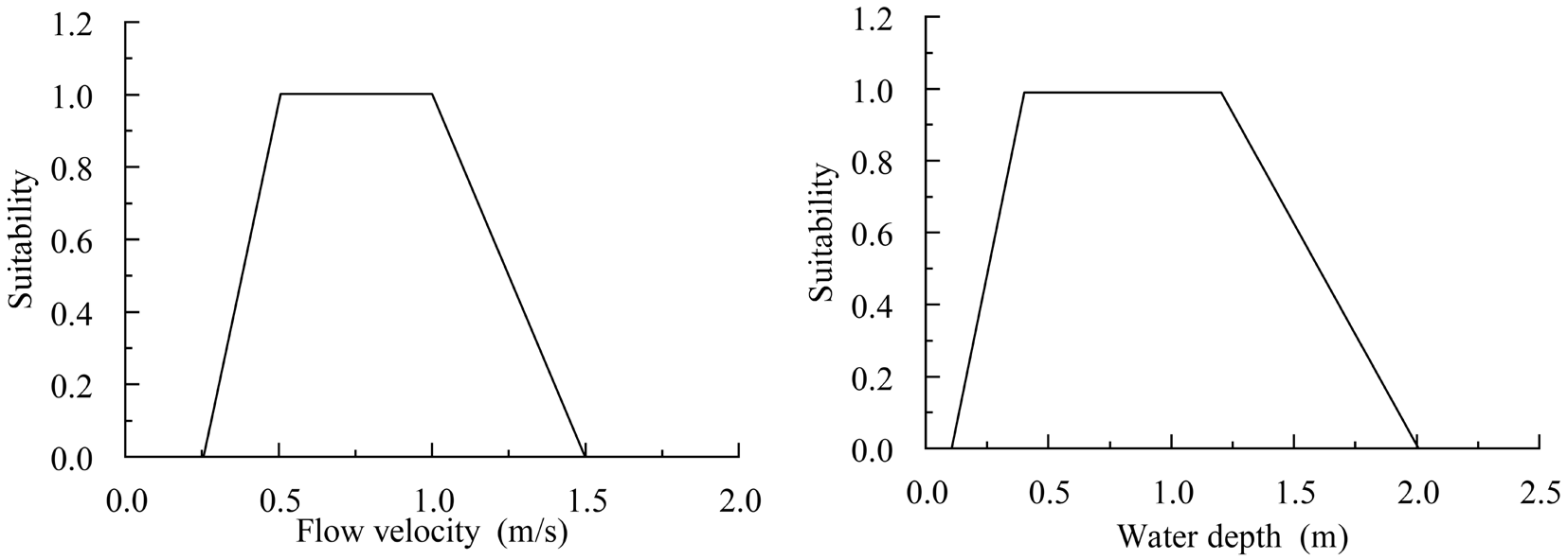
The adaptive curve of water depth and flow rate.

The upper reaches of the Yellow River basin encompassed seven key protected fish species. The developmental history of these fish could be broadly divided into several stages: the embryonic stage, the larval stage, the juvenile stage, the young fish stage, and the adult stage. Among these, the embryonic stage, larval stage, and juvenile stage were referred to as the early life history of fish, which was the most vulnerable and critical phase in their life cycle. The spawning period for important fish species occurs annually from April to July, requiring suitable spawning conditions such as water temperature, water depth, and attachment substrates for fish eggs. These species were batch spawners, and the breeding season generally lasted about 2 months. The spotted *Gymnocypris eckloni* inhabited wide valley rivers or lakes, primarily feeding on diatoms, cladophora, and chironomid larvae, while also consuming other foods such as rotifers and filamentous algae. The ovaries of the spotted *Gymnocypris eckloni* began developing in March, with the reproductive season occurring from April to May, occasionally extending into June or July. Late May marked the peak spawning period. The spawning grounds for the spotted *Gymnocypris eckloni* were typically located in slow‐flowing areas or still waters along the banks of wide valley rivers, with a water depth of about 1 m, where the substrate consisted mainly of pebbles and gravel. Their fertilized eggs were slightly adhesive, sinking to the bottom and often adhering to the pebbles or gravel for incubation. Given that the study area falls within a broad river valley, conducive to the survival and reproduction of the *Gymnocypris eckloni* , it has been selected as the target species for this research (Quan et al. 2021; Guo et al. 2024). Therefore, *Gymnocypris eckloni* was selected as the target fish species for habitat simulation. Based on the survey results conducted by aquatic experts, Yang et al. (2021) collected and summarized the ecological habits and hydrological characteristics of *Gymnocypris eckloni* during its main spawning period. This led to the derivation of suitability curves for velocity and water depth. The peak range of the curves represented the optimal flow velocity and water depth ranges for *Gymnocypris eckloni* . As shown in Figure 2, the suitable water depth ranged from 0.1 to 2.0 m, with an optimal depth of 0.4 to 1.2 m. The suitable flow velocity ranged from 0.25 to 1.5 m/s, with an optimal velocity of 0.5 to 1.0 m/s.

### 2.3 Data Collection

#### 2.3.1 River Discharge Data

**FIGURE 3:**
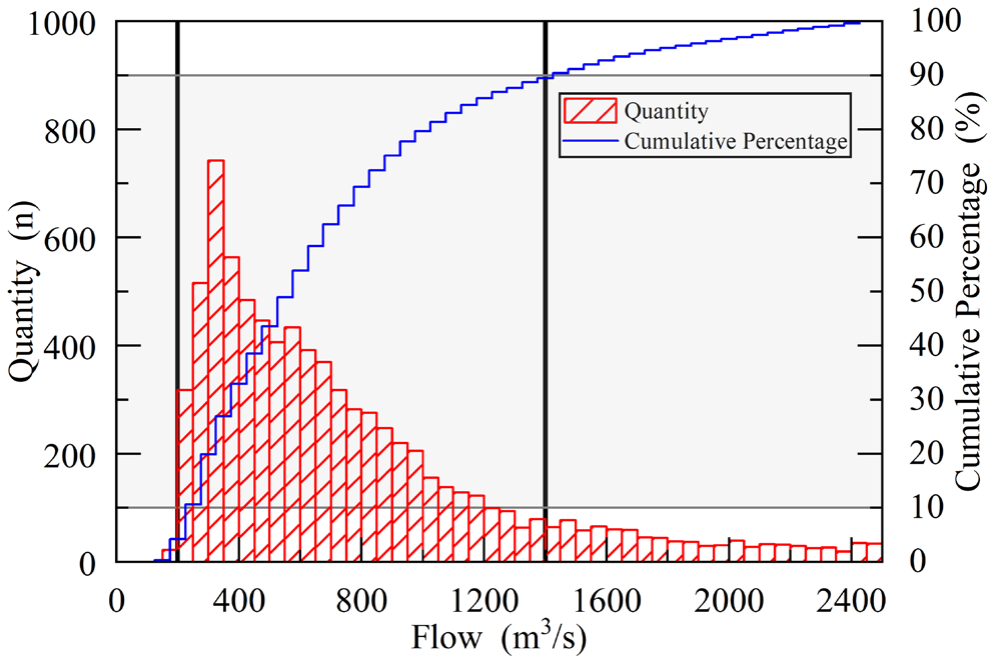
Statistical analysis of traffic during spawning periods from 1956 to 2022.

Statistical analysis was conducted on the hydrological data of the Tangnaihai Hydrological Station from 1956 to 2022. The natural inflow during the spawning period of *Gymnocypris eckloni* is shown in Figure 3. A flow range of 10%–90% within the natural inflow was selected as the simulation conditions. The minimum natural inflow was 200 m^3^/s, and the maximum natural inflow was 1400 m^3^/s. A set of conditions was established every 50 m^3^/s to cover all inflow scenarios during the spawning period within the statistical period, excluding extreme cases.

#### 2.3.2 Terrain Data of the Study Area

A 1:1000 terrain survey was conducted for the underwater terrain, waterfront, and overall riverbed of the study area. For underwater survey work, a multi‐beam echo sounder system was used for data collection. For exposed parts of the riverbed, Unmanned Aerial Vehicle (UAV)—mounted radar or oblique photography was used for data collection. For shallower water areas, a single‐beam echo sounder system was utilized for data collection. After the completion of data collection, terrain mapping was edited.

### 2.4 Research Methodology

A two‐dimensional hydrodynamic model was employed, coupled with a habitat model, to simulate the distribution of water depth and flow velocity in the river channel under different flow conditions. The adaptability of the target fish species, *Gymnocypris eckloni* , to its habitat, was analyzed, and the habitat was graded. A quantitative analysis was conducted to assess the impact of river flow variations on suitable habitats.

#### 2.4.1 Hydrodynamic Model

Based on the two‐dimensional shallow water equations of MIKE 21, a hydrodynamic model was constructed for the river section downstream of the Yangqu Hydropower Station. The governing equations for this model consisted of the continuity equation and the momentum equation.

Continuity Equation:

(1) $$\frac{\partial h}{\partial t}+\frac{\partial h\bar{u}}{\partial x}+\frac{\partial h\bar{v}}{\partial x}=\mathrm{hS}$$

*y*‐direction Momentum Equation:

(2) $$\begin{array}{l} \;\frac{\partial h\bar{u}}{\partial t}+\frac{\partial h{\bar{u}}^{2}}{\partial x}+\frac{\partial h\bar{u}\bar{v}}{\partial y}=f\bar{v}h-\mathrm{gh}\frac{\partial \eta }{\partial x}-\frac{h}{p_{0}}\frac{\partial p_{a}}{\partial x}-\frac{{\mathrm{gh}}^{2}}{2p_{0}}\frac{\partial p}{\partial x}+\frac{\tau_{\mathrm{sx}}}{p_{0}}-\frac{\tau_{\mathrm{bx}}}{p_{0}}-\frac{1}{p_{0}}\left(\frac{\partial s_{\mathrm{xx}}}{\partial x}+\frac{\partial s_{\mathrm{xy}}}{\partial y}\right)+\\ \;\frac{\partial }{\partial x}\left({\mathrm{hT}}_{\mathrm{xx}}\right)+\frac{\partial }{\partial y}\left({\mathrm{hT}}_{\mathrm{xy}}\right)+{\mathrm{hu}}_{s}S \end{array}$$

*y*‐direction Momentum Equation:

(3) $$\begin{array}{l} \;\frac{\partial h\bar{v}}{\partial t}+\frac{\partial h{\bar{v}}^{2}}{\partial y}+\frac{\partial h\bar{u}\bar{v}}{\partial x}=-f\bar{u}h-\mathrm{gh}\frac{\partial \eta }{\partial y}-\frac{h}{p_{0}}\frac{\partial p_{a}}{\partial y}-\frac{{\mathrm{gh}}^{2}}{2p_{0}}\frac{\partial p}{\partial y}+\frac{\tau_{\mathrm{sy}}}{p_{0}}-\frac{\tau_{\mathrm{by}}}{p_{0}}-\frac{1}{p_{0}}\left(\frac{\partial s_{\mathrm{yx}}}{\partial x}+\frac{\partial s_{\mathrm{yy}}}{\partial y}\right)+\\ \;\frac{\partial }{\partial x}\left({\mathrm{hT}}_{\mathrm{xy}}\right)+\frac{\partial }{\partial y}\left({\mathrm{hT}}_{\mathrm{yy}}\right)+{\mathrm{hu}}_{s}S. \end{array}$$

In the equation:

*t* represents time (*s*); *x* and *y* were the coordinates in the Cartesian coordinate system (*m*); *h* represents water level (*m*); *d* was the static water depth (*m*); *u* and *v were* the velocity components in the *x* and *y* directions (m/s); *f* was the Coriolis force coefficient; *ω* was the angular velocity of the Earth's rotation (rad/s); *ψ* was the local latitude; *g* was the acceleration due to gravity (m/s^2^); *ρ* was the density of water (kg/m^3^); *S* represents the source term; *u*_*s*_ and *v*_*s*_ were the flow velocities in the source term (m/s); *τ*_*sx*_, *τ*_*bx*_, *τ*_*sy*_, and *τ*_*by*_ were the components of surface wind stress and bed bottom friction stress along the *x* and *y* directions (kg m/s^2^).

#### 2.4.2 Nash‐Sutcliffe Efficiency (NSE)

The NSE was an important statistical metric used to assess the accuracy of predictive models, particularly in hydrological model validation. It quantified the degree of fit between the simulated and observed values, reflecting how well the model aligned with actual conditions (Peramuna et al. 2024). When NSE = 1, it indicated that the model's predictions were in perfect agreement with the observed data, representing optimal performance. When 0 ≤ NSE < 1, it suggested that the model performed well, though some errors existed. When NSE = 0, it meant that the model's performance was equivalent to using the mean of the observed values as a predictor, offering no additional improvement. If NSE < 0, it indicated that the model performed worse than simply using the mean of the observed values, reflecting very poor predictive capability.

(4) $$\mathrm{NSE}=1-\frac{\sum_{i=1}^{n}{\left(Q_{o}-Q_{m}\right)}^{2}}{\sum_{i=1}^{n}{\left(Q_{o}-{\bar{Q}}_{o}\right)}^{2}}.$$

In the equation:

*Q*_*o*_ represents the observed values; *Q*_*m*_ represents the model's predicted values; ${\bar{Q}}_{o}$ is the mean of the observed values; *n* is the number of observation data points.

#### 2.4.3 Fish Habitat Model

Habitat simulation relies on habitat suitability curves to obtain the combined suitability values for each unit's influencing factors. The overall microhabitat suitability of the study reach was calculated using Equation (5) and was referred to as the WUA

(5) $$\mathrm{WUA}=\sum_{i=1}^{n}\mathrm{CSF}\left(V_{i},D_{i},C_{i}\right)\times A_{i}.$$

In the formula:

WUA represents the microhabitat suitability area of the study reach (m^2^). *CSF*(*V*_*i*_, *D*_*i*_, *C*_*i*_) represented the combined suitability value of each unit's influencing factors (m/s), *D*_*i*_ was the water depth measured in meters (m), and *C*_*i*_ was the river index that included bed material and shelters. *A*_*i*_ was the projected area of each unit on the horizontal plane (m^2^).

The formula for habitat combined suitability value was as follows:

(6) $${\mathrm{CSF}}_{i}=\mathrm{MIN}\left(V_{i},D_{i},C_{i}\right).$$

#### 2.4.4 Fuzzy Logic Method

The assessment of fish habitat quality often involves various uncertain factors, such as water flow velocity, water depth, temperature, and water quality. The fuzzy logic method effectively handled these uncertainties by defining fuzzy sets (e.g., “Low,” “Medium,” and “High”) to describe environmental variables, thereby enhancing the understanding of how the environment influenced fish habitats (Dubos, St‐Hilaire, and Bergeron 2023; Kim et al. 2024). In previous studies, fuzzy logic methods were used to simulate flow velocity and water depth data for specific river reaches, serving as key indicators for evaluating fish habitat quality. By combining Python with the MIKE21 hydrodynamic model, researchers were able to extract fish distribution areas with varying habitat qualities.

The core of this methodology lay in the use of fuzzy sets to describe different states or grades of flow velocity and water depth, including “low velocity,” “medium velocity,” and “high velocity,” as well as “shallow water,” “medium water,” and “deep water.” To accurately represent each state, corresponding membership functions were designed for these fuzzy sets, with values ranging from 0 to 1, indicating the degree to which a particular data point belonged to a specific state. For instance, a certain value of flow velocity or water depth might partially belong to both “medium velocity” and “high velocity” states, and the membership function was used to quantify this relationship (Boavida et al. 2014; Pujaru et al. 2024). In this way, flow velocity and water depth data were systematically classified for further analysis. Before conducting fuzzy logic analysis, the flow velocity and water depth data generated by MIKE21 needed to undergo preprocessing to ensure compatibility with fuzzy logic. The preprocessing steps included data cleaning and format conversion to prepare the data for subsequent fuzzy analysis.

Based on the known ecological preferences of *Gymnocypris eckloni* regarding flow velocity and water depth, specific fuzzy rules were formulated. As shown in Table 1, when the membership degrees of both flow velocity and water depth were 1, it indicated that the habitat conditions were highly suitable for the species, and the habitat quality was evaluated as “High.” If only one parameter met the criteria while the membership degree of the other was low, the habitat quality was assessed as “Medium.” When both parameters had low membership degrees, the habitat quality was determined to be “Low.” If the membership degree of either parameter was 0, it indicated that the area was unsuitable for the species.

**TABLE 1:** Habitat quality assessment criteria.

	High	Medium		Low	Unsuitable		
Flow velocity membership	1	(0, 1)	1	(0, 1)	0	(0, 1)	0
Water depth membership	1	1	(0, 1)	(0, 1)	(0, 1)	0	0

## 3 Results

Based on historical inflow data and the results of hydrodynamic simulations, calculation scenarios were established to investigate the trend of changes in fish habitat areas under different inflow conditions. This revealed the relationship between the WUA and the proportion of different quality habitats and the flow response.

### 3.1 Model Validation

**FIGURE 4:**
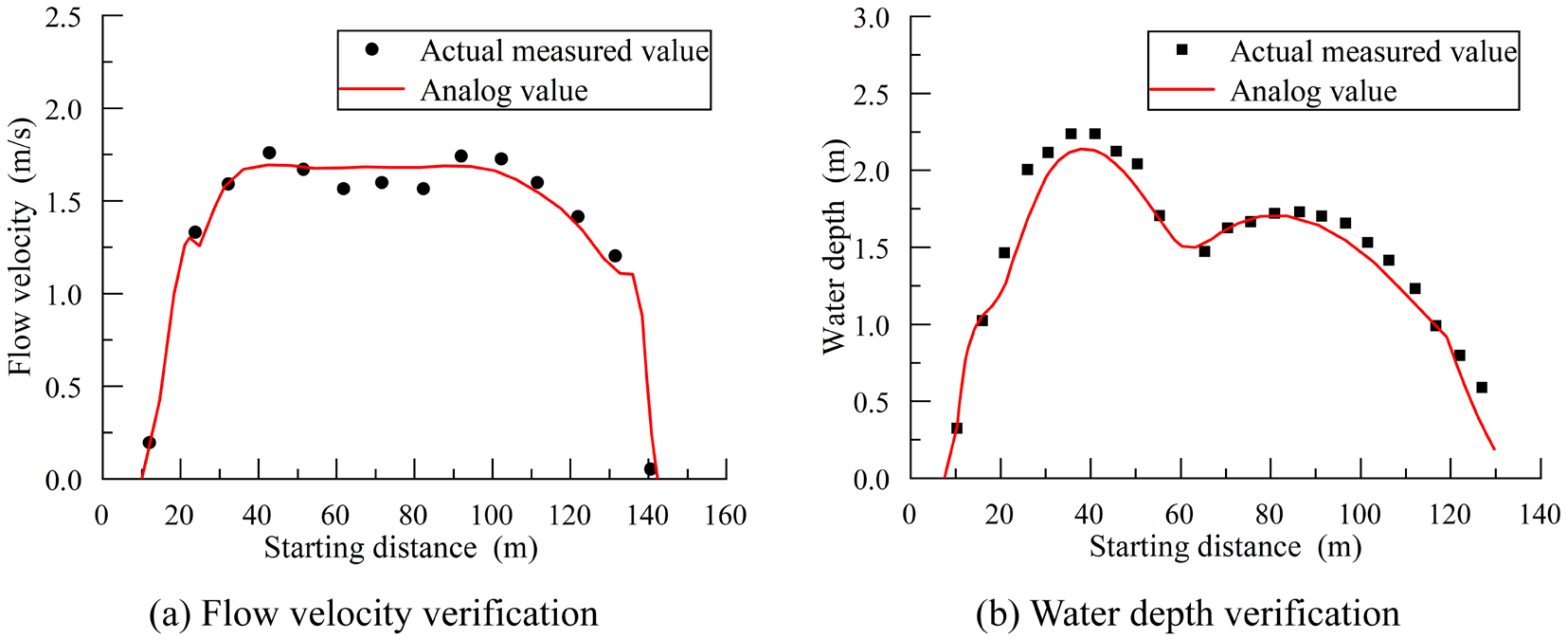
Model validation.

The model was meshed using unstructured grids, with local grid densification applied to the primary spawning areas of fish. A total of 79,481 unstructured grids were created for the model. In the target river section, the riverbed downstream of the dam was relatively gentle, primarily composed of gravel and pebbles, with a roughness coefficient (*n*) ranging from 0.025 to 0.029. In contrast, the river channel at the upper gorge entrance of the Yahu Gorge was narrow with a steep riverbed, and the roughness coefficient ranged from 0.035 to 0.04. The hydrodynamic numerical model provided fundamental data for the fish habitat model and adjustments were made to the model's roughness. Consequently, roughness coefficients of *n* = 0.029 and *n* = 0.035 were selected as the final roughness values for the target river section. The model's calculation results were compared and validated against measured values, as shown in Figure 4. From Figure 4, it can be seen that the model's calculation results agree well with the measured data. The maximum error between simulated and measured flow velocities was 5%, and the maximum error between simulated and measured water depths was 5.4%. The model's calculation results closely matched the measured data, yielding an NSE of 0.943. This indicates that the model has good predictive capability for water flow conditions in the study area, and the calculation results were reliable and accurate.

### 3.2 Hydrodynamic Characteristic Analysis

Based on the occurrence frequency of the daily average flow during the spawning period of the target fish species from 1956 to 2022, flow rates ranging from 10% to 90% were selected for the simulation conditions. The flow rate corresponding to the 10% frequency was 200 m^3^/s, defined as the low flow condition; the flow rate corresponding to the 50% frequency was 500 m^3^/s, defined as the moderate flow condition; the flow rate corresponding to the 70% frequency was 800 m^3^/s, defined as the relatively high flow condition; and the flow rate corresponding to the 90% frequency was 1400 m^3^/s, defined as the high flow condition.

**FIGURE 5:**
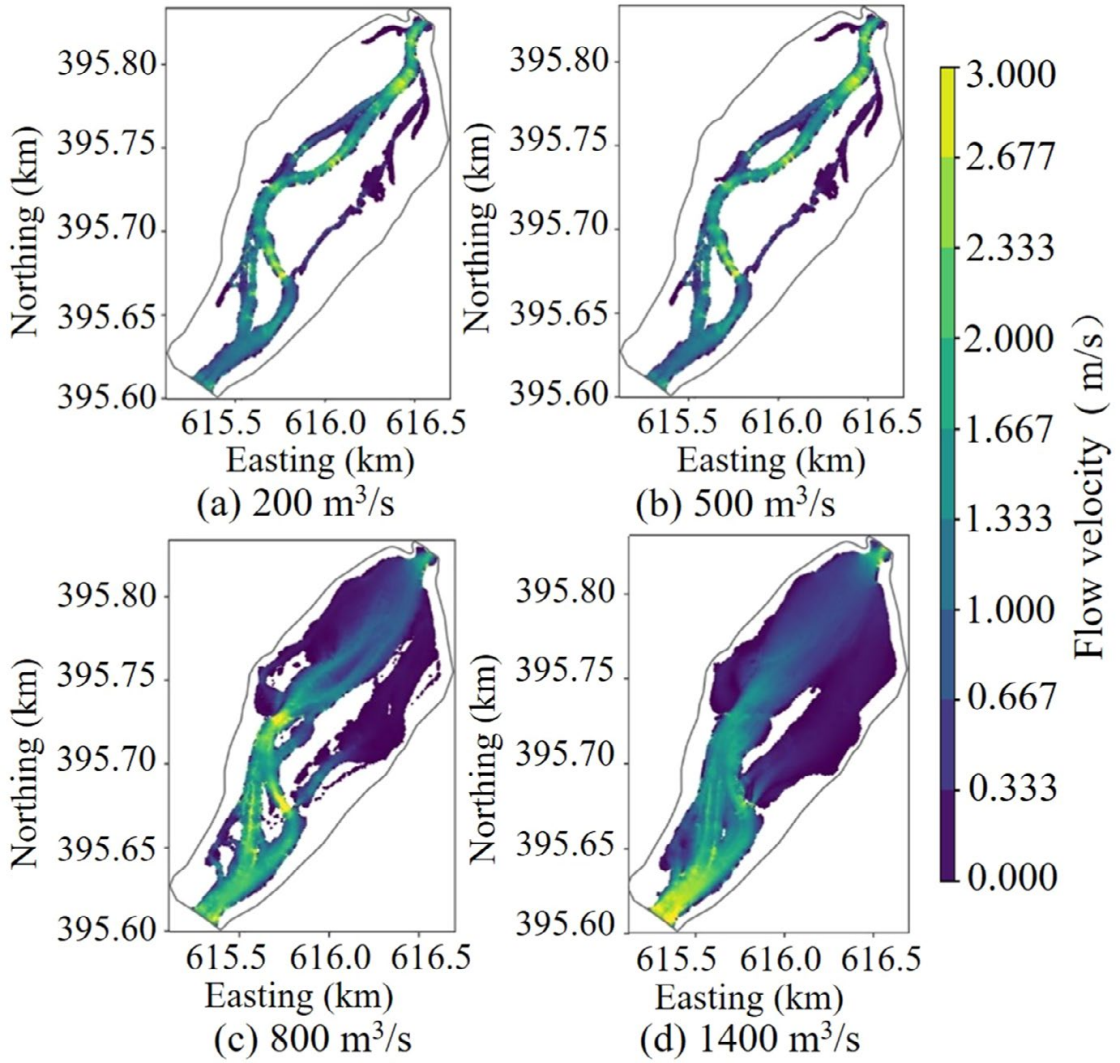
Flow velocity distribution under different flow states in the lower reaches of Yangqu Dam.

**FIGURE 6:**
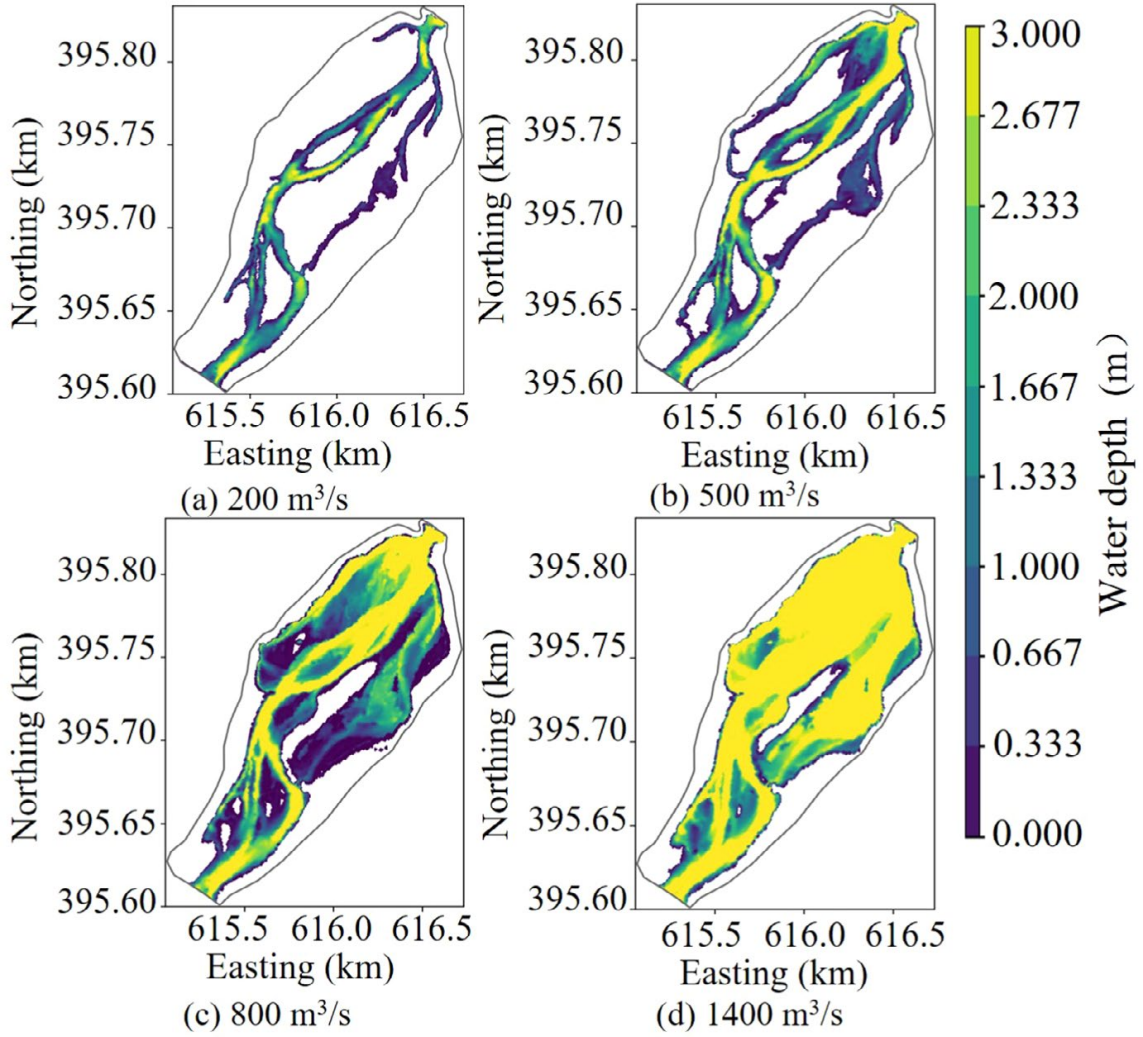
Water depth distribution under different flow states in the lower reach of Yangqu Dam.

Figures 5 and 6 were the velocity and depth distribution maps during low flow state (200 m^3^/s), medium flow state (500 m^3^/s), high flow state (800 m^3^/s), and very high flow state (1400 m^3^/s), respectively. During the low and medium flow rates, the river water primarily flowed within the main river channel, and a large area of backwater zones formed along the left bank of the upstream river channel and the right bank of the middle and downstream river channel. The flow velocity within the main river channel was less than 2.5 m/s, and the water depth was less than 3 m. In contrast, the flow velocity in the backwater zones was less than 0.3 m/s, and the water depth was less than 1.5 m. As the flow rate increased, under higher flow conditions, the downstream outlet began to back up water. This resulted in a significant rise in the downstream water level, a decrease in flow velocity, and extensive flooding of shallow beaches on both the left and right banks of the downstream area. The flow velocity in these areas was relatively low, while the water depth in the main river channel exceeded 3 m. In the upstream areas that were not affected, the flow velocity increased significantly, with local flow velocities exceeding 3 m/s. As the flow rate continued to increase, reaching the high flow state, the overall water level of the river channel rose. This led to the complete submergence of shallow beaches in the middle reaches with a water depth exceeding 3 m. The backwater zone reached its maximum extent, while the water depth in some shallow beaches upstream was less than 1.5 m.

### 3.3 Fish Habitat Suitability Distribution

**FIGURE 7:**
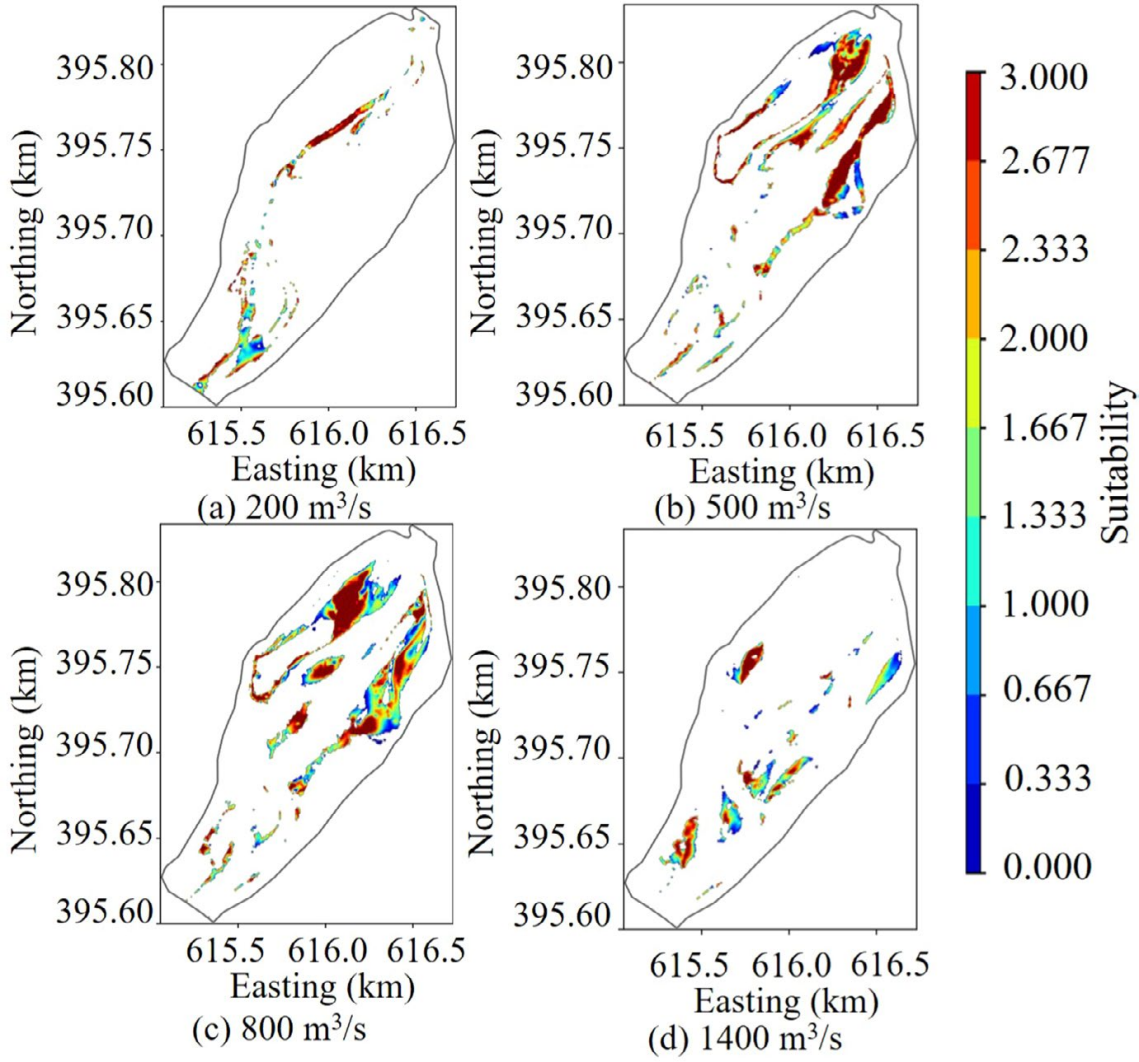
The regional distribution of different flow suitability of river sections was studied.

The analysis of habitat changes was conducted by selecting four flow states: low (200 m^3^/s), medium (500 m^3^/s), high (800 m^3^/s), and very high (1400 m^3^/s) in the studied river reach. Figure 7 illustrates the distribution of suitable habitat areas under different flow conditions in the studied river reach. Based on the comprehensive results of hydrodynamic calculations and habitat suitability assessments, it could be concluded that the area of suitable habitat for *Gymnocypris eckloni* first increased and then decreased as the flow rate increased. The spawning grounds were primarily distributed in the large backwater areas along the left bank of the upstream river channel and in the shallow beach areas of the middle and downstream river channel. At medium and high flow rates, most of the habitats in the shallow beaches of the middle and downstream river channel became submerged, except for certain areas preserved in the backwater bends along the left bank of the upstream river channel. This submersion resulted in the maximum habitat area for the target fish species. However, as the flow rate further increased to very high levels, the submersion depth of the shallow beaches exceeded the optimal range, reducing habitat suitability. Consequently, the primary spawning grounds shifted to localized backwater regions along the riverbanks, where conditions remained more favorable for the target fish species.

### 3.4 Relationship Between WUA of Quality Habitats and Flow Response

**FIGURE 8:**
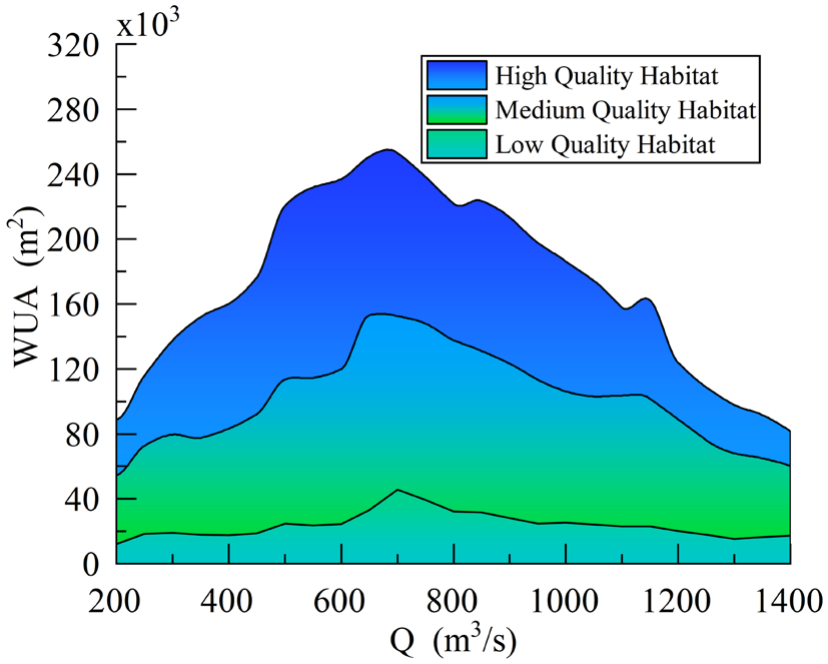
Relationship between WUA and flow change of different mass habitats.

The relationship between the WUA of different quality habitats for the target fish species and flow rate is shown in Figure 8. From Figure 8, it can be seen that as the flow rate increased in the river channel, the total weighted habitat area for the target fish species first increased and then decreased. The maximum area occurred when the flow rate reached 700 m^3^/s, totaling 253,046.89 m^2^. The minimum area occurred at a flow rate of 1400 m^3^/s, with only 81,739.42 m^2^. The WUA of high‐quality habitats was largest at a flow rate of 650 m^3^/s and smallest at 1400 m^3^/s. The WUA of medium‐quality habitats was largest at a flow rate of 650 m^3^/s and smallest at 200 m^3^/s. The WUA of low‐quality habitats was largest at a flow rate of 700 m^3^/s and smallest at 200 m^3^/s. The overall trend of the WUA for high‐and medium‐quality habitats was consistent, and both exhibited relatively large variations. In contrast, the WUA of low‐quality habitats exhibited a smaller range of variation underflow rate fluctuations, indicating that it was less influenced by changes in flow rate. Through analysis, the flow range corresponding to 60% of the total weighted habitat area was identified as the ecological flow requirement for the target fish species. This range, from 350 to 1100 m^3^/s, effectively balanced the maintenance of suitable fish habitats with the stability and diversity of the river ecosystem. The selection of this flow range comprehensively considered the response of habitats of varying quality to flow rate changes.

**FIGURE 9:**
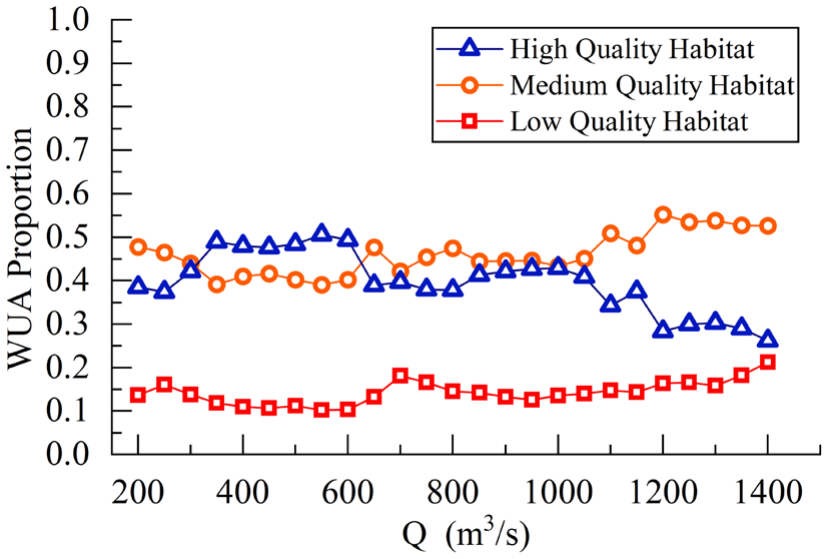
The relationship between the WUA ratio and the flow change of different quality habitats.

The relationship between the proportion of WUA of different quality habitats to the total area and flow rate changes is shown in Figure 9. From Figure 9, it can be seen that the WUA of low‐quality habitats remained relatively stable between 0.1 and 0.2, with a slight increase in its proportion of the total area. The WUA of high‐quality habitats increased from 0.4 to 0.5 within the flow rate range of 200 to 350 m^3^/s and then stabilized, reaching its maximum proportion at this time. When the flow rate increased to 600 m^3^/s, there was a slight decrease, followed by a slight increase as the flow rate continued to increase, remaining between 0.37 and 0.4 overall. When the flow rate was between 1050 and 1400 m^3^/s, the WUA of high‐quality habitats decreased significantly. The proportion of the WUA of medium‐quality habitats decreased slightly in the early stages of flow rate increase, but then stabilized. After the flow rate increased to 600 m^3^/s, the proportion of the area gradually increased, becoming the largest proportion of the total area. When the flow rate increased to 1050 m^3^/s, the proportion of the area increased significantly and stabilized around 0.5 after the flow rate increased to 1200 m^3^/s. Therefore, an interval with relatively small differences in the proportions of medium‐ and high‐quality habitat WUA, namely 600 to 1150 m^3^/s, could be selected as a reference for the ecological flow requirement interval for the target fish species.

**FIGURE 10:**
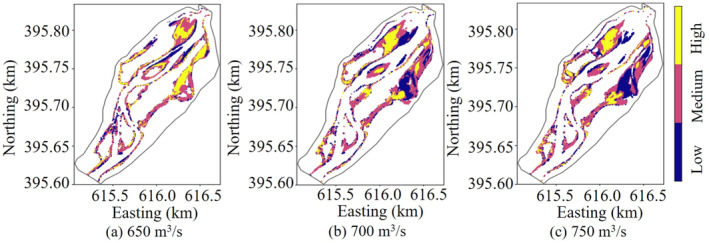
Cloud map of the maximum WUA of high, medium and low mass under different flow rates.

Figure 10 shows the distribution of WUA for high, medium, and low‐quality habitats when each type of habitat reaches its maximum value. As seen in Figure 10, when the WUA of high‐quality habitats was at its maximum, they were mostly distributed in the backwater areas along the left and right banks of the upstream river channel. As the flow rate gradually increased, the backwater areas were submerged, resulting in a significant decrease in high‐quality habitats on the right bank, while the area of high‐quality habitats increased in the middle and downstream sections of the target river reach. When the WUA of medium‐quality habitats reached its maximum, these habitats were widely distributed in both the backwater zones and the main river channel. Notably, as the flow rate fluctuated, the spatial distribution of medium‐quality habitats remained nearly unchanged, indicating their high stability and relatively low susceptibility to flow rate variations compared to high‐quality habitats. In contrast, low‐quality habitats were predominantly located in the backwater areas and shallow shoals along the banks of the upstream section, with occasional scattered distribution in the downstream gorge. The locations of these habitats were relatively fixed, and their total area was minimally affected by flow rate changes. This suggested that low‐quality habitats exhibited stronger adaptability to flow rate fluctuations, maintaining a consistent distribution pattern.

Through the analysis of habitat‐WUA changes with flow rate and the proportion of different quality habitat‐WUA to the total area, it was concluded that high‐quality habitat‐WUA was the most sensitive to flow rate variations in the target river reach, while low‐quality habitat‐WUA exhibited minimal sensitivity. This highlighted the importance of prioritizing both high‐ and medium‐quality habitat‐WUA when determining the minimum ecological flow. Based on a comprehensive analysis of the ecological flow range for the target fish species, which considered the habitat‐WUA and the proportional changes in different quality habitats, it was observed that significant variability occurred in the ratio between medium‐ and high‐quality habitat‐WUA within the flow range of 350 to 600 m^3^/s. Additionally, at a flow rate of 1100 m^3^/s, the total habitat‐WUA reached 162,319.65 m^2^, accounting for 64% of the maximum total habitat area, making this flow rate suitable as the maximum ecological flow for the target species. Considering these findings, the study recommended a flow rate range of 600 to 1100 m^3^/s as the ecological flow interval for the target fish species in the studied river reach. This range effectively balances the preservation of high‐ and medium‐quality habitats while ensuring sufficient ecological support for the species.

## 4 Discussion

### 4.1 The Impact of Dam Construction on Fish Habitats

The study area was located downstream of the Yangqu Hydropower Station dam and upstream of Yehu Gorge, a narrow cliff canyon that had formed an extensive backwater zone, providing a natural habitat for fish species. However, the construction of the dam significantly altered the original river flow conditions, limiting the ability of fish to freely select habitats for foraging and spawning. It disrupted the natural hydrological cycle and interrupted river channel connectivity (Zhang et al. 2020; López‐Rodríguez et al. 2024). Despite the implementation of protective measures, such as fish ladders, the original aquatic ecosystem remained notably impacted. The dam's construction changed the river's flow velocity, water depth, temperature, substrate, and topography. These changes not only affected fish survival behaviors but also disrupted essential physiological functions (Lin et al. 2021; Yang et al. 2023). Therefore, studying the relationship between habitat area, habitat quality, and flow variations in this region became necessary to mitigate the effects of hydropower construction on fish habitats and support ecological restoration efforts.

### 4.2 Evaluation of Habitat Suitability Model Based on Fuzzy Logic

**FIGURE 11:**
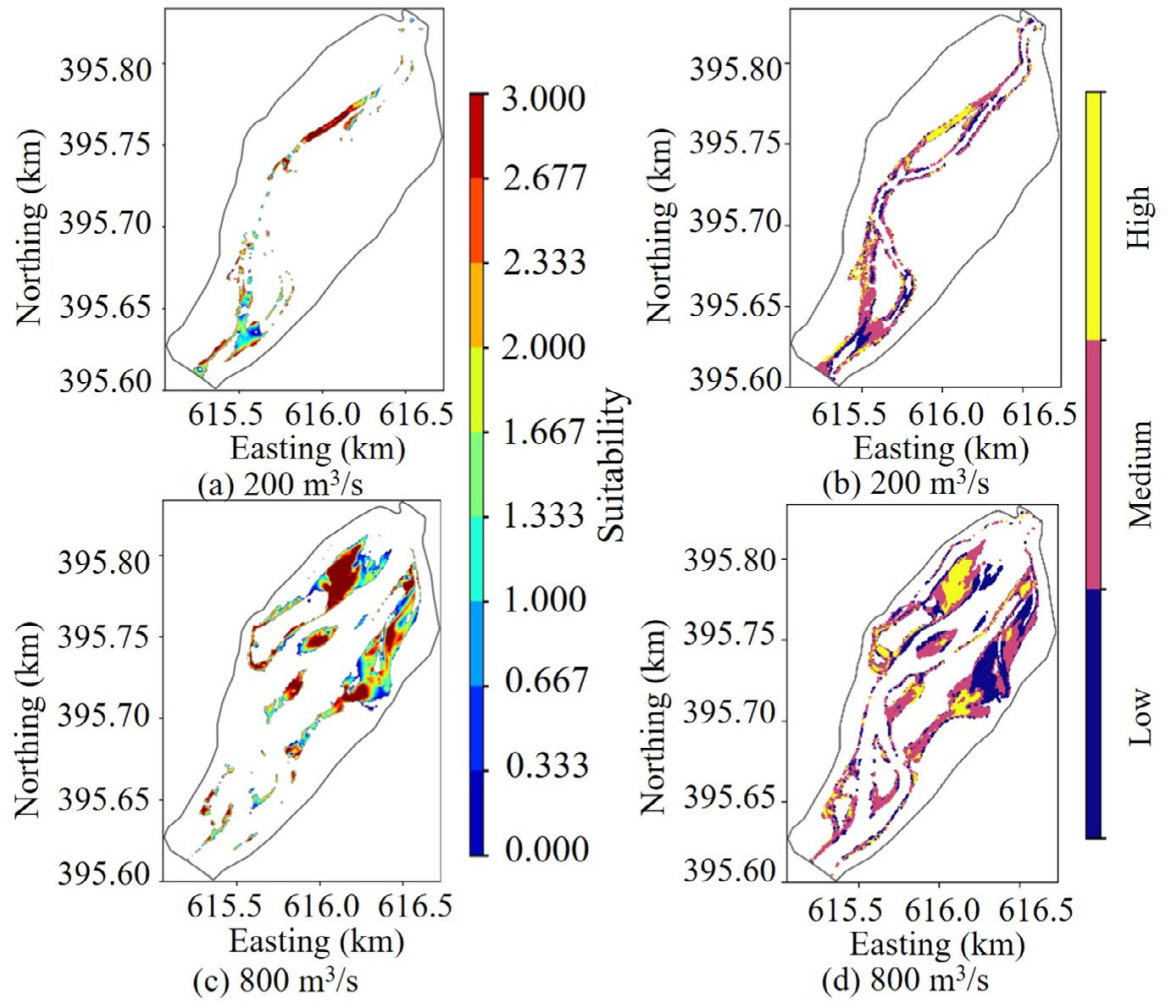
Habitat distribution of traditional habitat simulation versus the fuzzy logic‐based method.

The traditional habitat model only considered the size of the fish habitat area, ignoring the importance of habitat quality (Wang, Li et al. 2023). This study aimed to address this limitation by measuring the river channel morphology after the construction of water conservancy projects and developing a more accurate fish habitat model (Kim and Choi 2021; Zhao et al. 2024). Based on the habitat simulation method, fuzzy logic was introduced to classify habitat quality based on suitable flow velocity and water depth for the target fish species. This approach established a relationship between the WUA of different quality habitats and flow rate changes. As shown in Figure 11, images (a) and (c) represented the habitat suitability distribution derived from the traditional habitat simulation method under flow conditions of 200 and 800 m^3^/s, respectively. In contrast, images (b) and (d) illustrated the habitat quality distribution obtained using the fuzzy logic method under the same flow conditions. The habitat suitability area distributions produced by both methods were generally similar; however, the traditional habitat simulation method provided a more detailed variation in suitability areas within the river reach, albeit with poorer connectivity between patches. In comparison, the fuzzy logic method systematically classified flow velocity and water depth data, which allowed for a clearer analysis of habitat quality distribution and highlighted the differences in habitat quality more distinctly (Ouellet et al. 2021; Çelekli et al. 2024). The habitat suitability model based on fuzzy logic assigned index weights more scientifically and effectively evaluated the suitability of fish habitats.

### 4.3 Analysis of the Impact of Habitat Quality Distribution on Ecological Flow Optimization

As shown in Figure 8, selecting a flow range corresponding to 60% of the total weighted habitat area as the ecological flow required for the target fish species resulted in a broader flow range. However, Figure 9 revealed significant differences in the proportion of WUA of different quality habitats under low flow conditions, indicating that these flow levels were not suitable as ecological flows for the target river reach. Additionally, as the flow rate increased, high‐quality habitats gradually decreased, while the total habitat area first increased and then decreased, suggesting that high‐quality habitats should not serve as the primary basis for ecological flow selection. In contrast, low‐quality habitats were less affected by flow rate changes and only needed to maintain a stable proportion. Therefore, medium‐quality habitats should have been the primary criterion for determining ecological flow. By comprehensively analyzing the relationship between habitat WUA and the flow rate changes in different quality habitats, the ecological flow range for the target fish species obtained was more aligned with the actual survival needs of the fish. This approach avoided biases in ecological flow estimation caused by solely considering habitat area, thus ensuring the feasibility and effectiveness of habitat protection measures.

## 5 Conclusions

In this study, the MIKE21 hydrodynamic model was coupled with a fish habitat model to evaluate the suitability of habitats for the target fish species within the study area. The fuzzy logic method was used to classify habitat quality, and the analysis focused on the WUA and the proportion of different quality habitats. A response relationship between WUA, habitat quality proportions, and flow rate was established. The main conclusions drawn from this study were as follows:

1. The ecological flow range in the studied river section was determined to be between 600 and 1100 m^3^/s. Within this range, the habitat‐WUA was maximized, and there was a minimal difference in the proportion of medium‐ and high‐quality habitat‐WUA. Under these conditions, the habitat‐WUA was less affected by flow rate variations, making it more suitable for fish survival.
2. As the flow rate increased, the proportion of WUA of different quality habitats varied. The high‐quality habitat‐WUA gradually transitioned to medium and low‐quality habitat‐WUA, with medium‐quality habitats gradually becoming the largest proportion. The proportion of high‐quality habitat‐WUA ranged from 0.26 to 0.50, increasing first and then decreasing with increasing flow rate, making it the most affected by flow rate variations. The proportion of medium‐quality habitat‐WUA fluctuated between 0.40 and 0.55, showing an overall increasing trend, while the proportion of low‐quality habitat‐WUA increased from 0.11 to 0.21, being the least affected by flow rate variations.
3. The habitats of the target fish species were distributed in the large backwater area on the left bank of the upstream river section and a certain area of shallow water regions in the middle and downstream river sections. As the flow rate increased, the submerged depth of the shallow water areas increased, reducing their suitability, and the main spawning ground shifted to localized backwater areas along the riverbank.

## Author Contributions

**Qiaoling Zhang:** conceptualization (equal), methodology (equal), project administration (equal), writing – original draft (equal). **Youjie Ou:** conceptualization (equal), writing – original draft (equal). **Weiying Wang:** conceptualization (equal), funding acquisition (equal), investigation (equal). **Guoyong Zhang:** investigation (equal), supervision (equal), validation (equal). **Zijun Liu:** data curation (equal), methodology (equal). **Shanshan Li:** funding acquisition (equal), visualization (equal). **Guodong Li:** funding acquisition (equal), project administration (equal).

## Ethics Statement

The authors have nothing to report.

## Conflicts of Interest

The authors declare no conflicts of interest.

## Data Availability

The data for this paper have been uploaded to http://datadryad.org/stash/share/vIKcf8cEzgmNKa7hLvvLxf3t05n7‐viHBKLh6YSCSYU.
